# Lack of Food Standards

**Published:** 1924-11

**Authors:** C. W. Hutt

**Affiliations:** Medical Officer of Holborn


### THE LACK OF FOOD STANDARDS.

### WHAT OTHER COUNTRIES ARE DOING.

By C. W. HUTT, M.A., M.D., D.P.H., Medical Officer of Holborn.

During the last two or three years especially considerable dissatisfaction has been expressed at the absence of adequate legislation to ensure the purity and quality of food. As a consequence, the Departmental Committee on the use of preservatives and colouring matters in food was reappointed in July of last year; its final report is eagerly awaited by those interested. But unless its recommendations are made into law the position of local authorities concerned with the administration of the Sale of Food and Drugs Act will be but little improved. If, and when, the matter is taken up by Parliament it would greatly facilitate matters if a new Sale of Food and Drugs Act were passed which would not only codify the whole of the laws relating to the preparation, sale and storage of food, but would include such definitions of various articles of food as would more or less settle finally, as far as local authorities are concerned, the many questions outstanding at the present time.

### A Codex Alimentarius.

When these things are done the experience of Austria will have to be taken into account. In 1907 an official commission was appointed consisting of representatives of the Ministers concerned, the chief health officials, professional men and one member of the Chamber of Commerce of Vienna. From these a small sub-committee was appointed of professional men, health officials, a representative of the Viennese Chamber of Commerce, and a small number of experts chosen from manufacturers and traders; these latter varied according to the articles of food under consideration. The *Codex Alimentarius* thus formulated did not, however, obtain the force of law. The analysts were required to use the methods of examination laid down, and in forming their conclusions to pay regard to the standards given in the Codex. But the courts of justice were not required to base their verdicts entirely on the Codex; to the magistrates it is only an official collection of the opinion of experts which serves as a guide. They are at liberty to use their own judgment when considering their judgment. The institution of the Codex has had the effect of diminishing almost to vanishing point complaints of unfairness of legal verdicts, and although some of the requirements are stringent, the Austrian trading world has come to see that they make for fair dealing between vendor and consumer.

### Standards in the Dominions.

The formation of the Austrian Codex has had an effect, direct and indirect, on the food legislation of many other countries. Although they do not follow the Codex closely, the Sale of Food and Drugs Acts and the Regulations made under them by Canada, New Zealand and the several Australian States, New South Wales, Victoria, Queensland, South Australia, Western Australia and Tasmania all include standards. These countries have gone farther than Austria in that these are legal standards. The requirements of each of these States, although in the main similar, are by no means identical. As a rule the Regulations do not enact that certain tests shall be followed in the analysis, a marked feature of the Austrian Codex consequent on the history of its origin. However, the Regulations for South Australia do enact in the case of the determination of fat in milk the mode of sampling to be followed under varying conditions and lay down the method of carrying out the test.

### Pure Bread.

In this country controversies on the correct terminology of the various kinds of flour and bread have been going on for many years. Obviously in the case of a foodstuff which constitutes so large a part of the daily food of millions the public should be in a position to know exactly what they are eating. Any manipulation of flour, such as bleaching, which might permit an inferior quality to be sold as a superior article, is undesirable. The Regulations under the New Zealand Sale of Food and Drugs Act, 1908, for example, make the position quite clear. In them flour is distinguished as wholemeal flour, part wholemeal flour, flour, and self-raising flour. They require that wholemeal flour shall be the clean, sound product obtained by grinding and milling well cleaned, sound, milling wheat, and shall contain all the constituents of such wheat (including the germ); it shall contain not more than fourteen parts per cent. of moisture. Mixtures of flour and bran must not be sold as wholemeal flour. Flour must not be artificially bleached, nor shall it be sprayed at any stage of its production. Various problems which in England are only in the stage of expressions of opinion in reports by officials of the Ministry of Health, are boldly and clearly legislated upon in New Zealand. For instance, the Regulations require that flour shall not contain any added substance; in other words, no "improvers," such as calcium phosphate and sodium persulphate, may be added to flour so as to enable the baker to bake a loaf containing a higher proportion of water and thus to make more loaves from a sack of flour.

### Regulating the Sausage.

Much fraud goes on in connection with sausages in this country; in the absence of a definition a large quantity of a less expensive food such as bread can be added to a small quantity of meat and the mixture sold at a price approximating to that of meat of fair quality. The New Zealand Regulations require that minced meat, sausage-meat and saveloy sausage-meat shall be chopped or comminuted meat, with or without salt, sugar, spices, herbs, saltpetre (potassium or sodium nitrate) and wholesome farinaceous substances. It shall contain not less than 75 per cent. of meat of the kind or kinds designated on the label attached to the outside of the package in which it is contained, nor more than fourteen grains of saltpetre (potassium or sodium nitrate, calculated as potassium nitrate) to the pound, provided that if minced meat, sausage-meat and saveloy sausage-meat be sold enclosed in a skin of animal origin the said skin shall be deemed to be an integral portion of the said meat.

### The English Lack of Standards.

The only substances with regard to which power to make standards exists in this country are milk, cream and cheese, also butter and milk-blended butter in so far as the proportion of any milk solid other than milk fat is concerned. In other words, the amount of water permissible may be limited. The Ministry of Agriculture has issued standards for milk and butter; very indirectly a standard for cream is given in the Public Health (Milk and Cream) Regulations, 1912. The Ministry of Agriculture has not found itself in a position to use its powers to make standards with regard to cheese, yet standards with regard to hard cheese and cream cheese are badly wanted. We are in the same weak position as Austria before the issue of the Codex; it is left to an enterprising local authority administering the Sale of Food and Drugs Acts to undertake expensive prosecutions with every prospect, in the event of success, of a more expensive appeal to a higher court. The Ministry of Health can only report that "fines were also imposed in respect of the sale as Cheshire cheese of two samples containing respectively 31 per cent. and 33.3 per cent. only of fat calculated in the moisture-free content, whereas it is generally considered that Cheshire cheese and cheese of similar character should contain at least 45 per cent. of fat calculated on this basis." And again, "although the conviction in this case (brought by the Metropolitan Borough of Holborn) was quashed on appeal on a technical point, the conclusion on which the conviction was based—viz., that cheese made from separated or partly separated milk should not be described as cream cheese—does not appear to have been disputed."

### The Example of New Zealand.

Contrast the definite position established by the issue of standards. The New Zealand Regulations state that "Cheese is to contain in the water-free substance not less than fifty parts per cent. of fat wholly derived from milk and it shall not contain any foreign fat. Skim-milk cheese is to contain not less than ten parts per cent. of fat wholly derived from milk. Cream cheese shall be cheese made from milk and cream or from milk containing not less than six parts per cent. of milk-fat. It shall contain in the water-free substance not less than sixty parts per cent. of fat wholly derived from milk." Similar instances could readily be given of the value of standards in respect of articles of food, notably cocoa and allied substances, pure fruit cordials and syrups, baking powder, self-raising flour, custard powders, infants' foods, vinegar, honey and jam.

### The Coming Opportunity.

A Government Bill to set up standards of quality and purity of foods was submitted to the House of Commons in 1913, but as it was far from an agreed measure failed to pass. In 1914 the President of the Local Government Board, on being asked when he proposed to reintroduce the Bill, replied, "When one has an opportunity." It is to be hoped that soon after the final report the Departmental Committee on Preservatives and Colouring Matters in Food is issued, the task of introducing this much wanted legislation will be once more taken up.

